# *Candida glabrata*: A Unique Cause of Necrotizing Urethritis

**DOI:** 10.1155/2018/5263438

**Published:** 2018-06-05

**Authors:** Christine Ibilibor, James T. Cammack

**Affiliations:** Department of Urology, Texas Tech University Health Sciences Center, Lubbock, TX, USA

## Abstract

Necrotizing urethritis is a rare malady with only one other case reported in the literature found to be due to an infectious cause. We report a case of necrotizing urethritis caused by *Candida glabrata* and review all relevant literature to date. The patient is a 56-year-old man with a past medical history significant for poorly controlled insulin-dependent type 2 diabetes mellitus and incomplete bladder emptying who presented to the University Medical Center with perineal pain, fever, and urinary retention. Cross-sectional imaging showed emphysematous changes in the bulb of the corpus spongiosum. After admission, his fever and leukocytosis persisted, and his physical exam worsened with intravenous antibiotics alone. Subsequently, the patient underwent cystourethroscopy with incision and debridement of the corpus spongiosum. Postoperatively, he improved clinically and his spongiosum wound and urine grew *Candida glabrata*. To our knowledge, we report the first case of necrotizing urethritis caused by *Candida glabrata*.

## 1. Introduction

Necrotizing infection of the corpora cavernosa is rare and generally provoked by trauma, urethral instrumentation, or penile injection while abscess of the corpus spongiosum has been described in the setting of a rectal malignancy [1, 2]. However, necrotizing urethritis is exceedingly rare as there are only two other reported cases to our knowledge in the literature one of which was due to an infectious cause while the other was due to a Wegener's granulomatosis flare [3, 4]. Thus, we report a case of necrotizing urethritis caused by *Candida glabrata* and review all relevant literature to date.

## 2. Case Presentation

**Figure 1:**
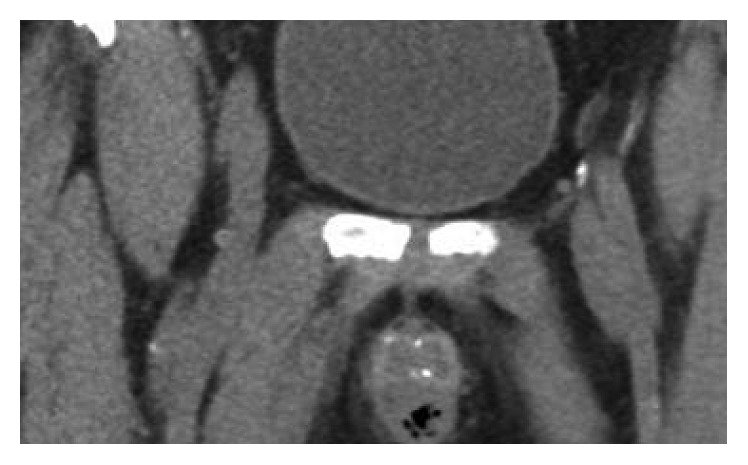
Coronal view of pelvic CT image demonstrating emphysematous changes in the bulb of the corpus spongiosum.

**Figure 2:**
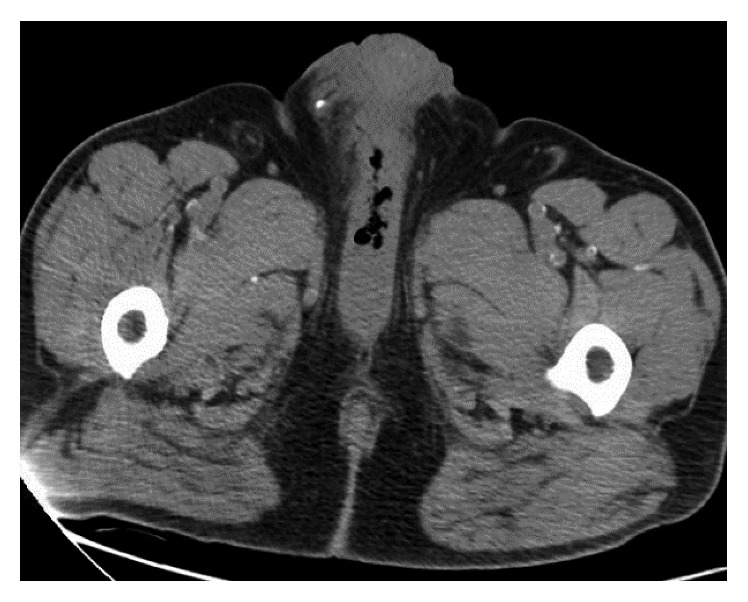
Axial view of pelvic CT image demonstrating emphysematous changes in the bulb of the corpus spongiosum.

The patient is a 56-year-old man with a past medical history significant for poorly controlled insulin-dependent type 2 diabetes mellitus, hypertension, hypothyroidism, and meatal stenosis with incomplete bladder emptying who presented the University Medical Center emergency room with a seven-day history of perineal pain and fever. One week prior to presentation, he was seen in the emergency room with the same symptoms and was placed on a 14-day course of ciprofloxacin for a suspected urinary tract infection. Also, the patient was admitted to the medical intensive care unit 10 months previously for severe sepsis secondary to *Candida glabrata* urinary tract infection with candidemia. The patient denied penile trauma, gross hematuria, and dysuria. Upon presentation, he was hemodynamically stable with a blood pressure of 145/77 mmHg, and he had mild tachycardia with a heart rate of 110 beats per minute and a fever of 101.4 F. On physical exam, the patient had mild tenderness to palpation at the penoscrotal junction with induration, no crepitus was palpated, and there were no skin changes. His laboratory values were significant for an elevated white blood cell count (WBC) of 20,000/*μ*L, a creatinine level of 1.4 mg/dL, and a hemoglobin A1c level of 9.4%, and all other values were within normal limits. On computerized tomography (CT) scan, emphysematous changes were noted with in the ventral portion of the penile shaft with air at the bulb of the corpus spongiosum (Figures 1 and 2).

**Figure 3:**
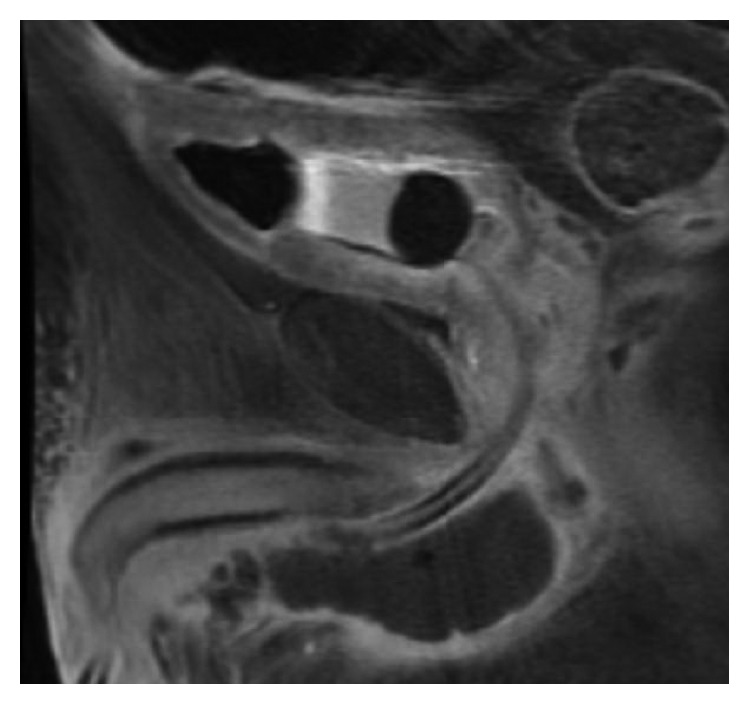
Sagittal view on MRI T1 image with gadolinium demonstrating decreased signal intensity in the corpus spongiosum compared to the corpora cavernosa consistent with reduced blood flow.

The patient had a postvoid residual of 320 ml; thus, a transurethral Foley catheter was placed with withdrawal of 700 ml of clear yellow urine which was sent for culture. The patient was begun on intravenous (IV) meropenem, fluconazole, and daptomycin. Repeat CT scan on hospital day 1 was largely unchanged, and the patient remained febrile to 102.3 F. On hospital day 2, meropenem and daptomycin were discontinued, and the patient was switched to piperacillin/tazobactam based on recommendations from the Infectious Disease team. A pelvic magnetic resonance imaging (MRI) with and without gadolinium was obtained at this time which showed reduced blood flow to the corpus spongiosum compared to the corpus cavernosum on T1-weighted imaging with gadolinium (Figure 3).

The patient was then taken to the operating room on hospital day 3 due to worsening penile pain and induration, a persistently elevated white blood cell and continued fever. On cystourethroscopy, the urethral mucosa appeared dusky from the anterior to the membranous urethra. A longitudinal incision was made in the perineum, the urethra was palpated and noted to be indurated and firm, and thus the bulbospongiosus muscle was split and a longitudinal incision was made in Buck's fascia with expulsion of approximately 10 ml of purulent fluid which was sent for culture and gram stain. The remaining necrotic spongiosal tissue was debrided with blunt dissection, a penrose drain was placed within the defect, and the skin was closed loosely. On postoperative day 2, his white blood count decreased to 13,000/*μ*L and he remained afebrile for 48 hours. The patient's intraoperative wound culture was found to be positive, and using automated Matrix Assisted Laser Desorption Ionization Time of Flight (MALDI-TOF) mass spectrometry, the organism *Candida glabrata* was identified by our microbiology laboratory. The wound culture result was identical to his urine culture. The patient was then switched to micafungin based on the Infectious Disease team's recommendations. He was subsequently discharged to home on postoperative day 14 on a 14-day course of oral voriconazole with the transurethral Foley in place.

## 3. Discussion

Immunocompromised patients such as those with poorly controlled diabetes mellitus are exceedingly susceptible to opportunistic pathogens such as *Candida glabrata* [5, 6]. In addition, *Candida glabrata* has been implicated as the casual pathogen in necrotizing soft tissue infections and lower urinary tract infections in the immunocompromised [7]. Loulergue et al. reported a case of Fournier's gangrene in a diabetic patient caused by *Candida glabrata* [5]. While a necrotizing infection of the spongiosal tissue is rare, the presence of *Candida glabrata* as the causal pathogen in the case presented here is not unexpected due to the patient's immunocompromised state and his prior admission for urosepsis due to *Candida glabrata*. *Candida glabrata* is a nondimorphic fungus that unlike other *Candida* species does not form pseudohyphae and has been known to cause urinary tract infections in elderly hospitalized patients with indwelling catheters and systemic infection in the immunocompromised [7]. With the increased prevalence of azole-resistant *Candida glabrata*, this case demonstrates the utility of beginning micafungin empirically particularly in a patient with a prior history of *Candida glabrata* infection [8].

In a similar case, *Aerococcus urinae* was found to be the causal pathogen in a case of necrotizing urethritis reported by Babaeer et al. [3]. It is a gram-positive coccus that can colonize the urinary tract and has been implicated in severe infections in elderly patients with benign prostatic hyperplasia [9].

Although the mainstay in the management of necrotizing soft tissue infection is wide often repeated debridement, the patient presented here underwent incision and debridement of the necrotic periurethral tissue and his urethra largely remained intact. In contrast, the case of necrotizing urethritis reported by Babaeer et al. required total urethrectomy to effectively treat the patient [3]. We believe that this difference in surgical management is due to the difference in severity of the infection and changes noted on cystourethroscopy. Our patient was hemodynamically stable at presentation while the patient presented by Babaeer et al. was in septic shock, also the patient presented here had no signs of necrosis on urethroscopy while the opposite was true for the Babaeer et al. patient at the time of urethrectomy [3]. Thus, we present a case of a patient with necrotic changes restricted to the corpus spongiosum sparing the urethral mucosa due to infection by *Candida glabrata* managed with incision of Buck's fascia and debridement of the necrotic spongiosal tissue.
